# Impact of postoperative flurbiprofen axetil on renal function in spontaneously hypertensive rats

**DOI:** 10.3389/fphar.2025.1686334

**Published:** 2025-12-18

**Authors:** Liang Sun, Xiaoran Ma, Yuhui Jiang, Wanda Zhang, Yi Feng

**Affiliations:** 1 Department of Anesthesiology, Peking University People’s Hospital, Beijing, China; 2 Department of Anesthesiology, Peking University People’s Hospital Shijiazhuang Campus, Shijiazhuang People’s Hospital, Shijiazhang, China; 3 Arthritis Clinic and Research Center, Peking University People’s Hospital, Beijing, China

**Keywords:** flurbiprofen axetil, glomerular, hypertension, pain behavior, renal function, tubular

## Abstract

**Background:**

Flurbiprofen axetil (FA) is a nonsteroidal anti-inflammatory drug frequently used in postoperative analgesia. However, limited data exist regarding its impact on renal profiles. Here we determine the potential impact of postoperative FA on renal function in spontaneously hypertensive (SH) rats.

**Methods:**

A plantar incision model was established to mimic post-surgical pain in SH rats. Animals were randomly assigned to groups called vehicle (fat emulsion, 0.5 mL/d), low-dose FA (H12.5, 12.5 mg/kg/d), medium-dose FA (H25, 25 mg/kg/d) or high-dose FA (H50, 50 mg/kg/d). Analogously, 40 male Wistar Kyoto (WKY) rats were included as controls in groups called vehicle, C12.5, C25, and C50. The vehicle and different doses of FA were administered from the day of surgery through postoperative day 2 (POD2). Paw withdrawal thresholds (PWTs) were measured before and after incision. Urinary N-acetyl-β-D-glucosaminidase (NAG), serum creatinine (SCr), and Cystatin C (Cys C) were measured. Animals were sacrificed for renal pathology analysis on POD7.

**Results:**

PWTs exhibited a dose-dependent increase after postoperative administration of FA on POD1 and POD2, but with a ceiling effect at the medium dose in both SH and WKY rats. Compared with the vehicle group, the levels of SCr in the C50, H25, and H50 groups were significantly increased on POD2 and POD7 (all P < 0.01). The concentration of Cys C in C12.5, C25, and C50 groups, as well as H12.5, H25, and H50 groups on POD2, were increased in a dose-dependent manner (all P < 0.05), similar trend was detected in H25, and H50 groups on POD7. Urine NAG levels in the H12.5, H25 and H50 groups on POD2, as well as H25 and H50 groups on POD7 were elevated in comparison with the vehicle groups (all P < 0.05). Partial glomerular and tubular damage was found in the H25 group. Extensive renal impairments were observed in the C50 and H50 groups.

**Conclusion:**

Medium-dose FA effectively alleviates postoperative pain and achieves a ceiling effect in SH rats. FA decreases glomerular and tubular function to a certain extent in both normo- and hypertensive rats. Renal impairment is detectable earlier after surgery in hypertensive rats, even at the lowest FA dose applied here.

## Introduction

Multimodal analgesia is a crucial component of enhanced recovery after surgery. In particular, non-steroidal anti-inflammatory drugs (NSAIDs) help reduce the use of postoperative opioids and shorten hospital stay (Wang et al., 2024; Butkus et al., 2023). Flurbiprofen axetil (FA), a non-selective NSAID, is widely used for postoperative pain management because it can be targeted to the surgical incision and inflammatory site, effectively relieving postoperative pain in multiple surgical procedures (Huo et al., 2025). Many patients can tolerate NSAIDs; for example, an observational study of 201 osteoarthritis patients with normal renal function observed only small, subclinical increases in serum creatinine (SCr) levels after 52 weeks of continuous administration of S-flurbiprofen plaster (Otsuka et al., 2017). However, FA and other NSAIDs, especially when given continuously at high doses, may elevate risk of serious renal adverse outcomes in patients with risk factors (e.g., advanced age, heart failure, concurrent use of antihypertensive drugs) (Hong et al., 2025). In addition, NSAIDs should not be taken by patients with preexisting uncontrolled hypertension and/or renal malfunction. This poses a growing challenge in today’s aging society, in which hypertension is increasingly prevalent among surgical patients (Alamri et al., 2024). For patients with good blood pressure control and normal preoperative renal function, NSAID guidelines remain unclear, especially for FA.

Currently, the lack of accurate indicators presents a major obstacle for the evaluation of renal function. SCr is still considered one of the main indicators for clinical evaluation of glomerular filtration rate (GFR), since it is much simpler to assay than GFR directly. However, SCr is easily affected by a battery of confounding factors such as age, sex, diet, and inflammation (Budano et al., 2020). Under such circumstances, SCr cannot accurately indicate early, mild glomerular impairment. Serum cystatin C (Cys C) may be an alternative to SCr for the estimation of GFR, since it is confounded by fewer extraneous factors, especially in hypertensive patients (Zaitoun et al., 2024). As a complementary index, the urinary activity of N-acetyl-β-D-glucosaminidase (NAG), which is mainly distributed in the lysosomes of the proximal tubular cells, is one of the most sensitive and extensively used markers of renal tubular impairment (Pan et al., 2025).

We are unaware of studies monitoring renal function in hypertensive surgical patients receiving different doses of FA as postoperative analgesia. As a step toward this goal, we used Cys C and NAG indices to monitor glomerular and renal tubular function in spontaneously hypertensive (SH) rats treated with FA.

## Materials and methods

### Animals

Male SH rats and age-matched Wistar-Kyoto (WKY) rats (190–220 g; Beijing Weitonglihua Experimental Animal Technology, Beijing, China) were housed with a 12-h light/dark cycle. Food and water were supplied *ad libitum*. Animal room temperature was controlled at 20 °C–22 °C with relative humidity of 40%–60%. All experiments were approved by the Ethics Committee of the Institute of Peking University People’s Hospital (2016PHC035) and performed in strict accordance with national laboratory animal guidelines.

### Blood pressure monitoring and data collection

Systolic blood pressure (SBP) was measured by the tail-cuff method on the day before surgery (between 8:00 and 12:00 h) using a non-invasive sphygmomanometer (BP2010A, Beijing Softong Biotechnology, Beijing, China). Animals were pre-warmed in a cabinet at 32 °C for 20 min to enhance the pulsation of the tail artery. Blood pressure was recorded at least three times at intervals of at least 5 min, and the mean was taken to be baseline SBP. In addition, data were also collected on body weight and other parameters (e.g., body temperature, nutrition, responses to surroundings, respiratory rate).

### Plantar incision

Rats were initially anesthetized with 5% isoflurane (Baxter Healthcare, IL, United States) for 2–3 min, and subsequently maintained under stable anesthesia with 2% isoflurane. On the left hind paw, a 1-cm longitudinal incision up to the plantaris muscle was made and then properly sutured as described (Brennan et al., 1996). The wound was examined daily to detect potential infection. We planned to exclude from the study any rats that developed wound infection, but none did.

### Pain behavioral test

The paw withdrawal threshold (PWT) to mechanical stimulation was determined using a series of von Frey filaments (Stoelting, Wood Dale, IL, United States) (1, 1.4, 2, 4, 6, 8, 10, 15, 26 g) according to the Dixon formula (Dixon, 1980). Briefly, the rats were acclimated to the surroundings for 15–30 min, and the von Frey filaments were vertically and sequentially applied on the plantar surface of the ipsilateral hind paw. Each stimulation lasted for 4–6 s, and stimulation was delivered five times at a 5-min interval. If a positive response occurred (e.g., abrupt paw withdrawal, licking, or shaking), the next smaller von Frey hair was used; if a negative response was observed, the next higher force was used. The test continued until (a) the responses to five stimuli were assessed after the first crossing of the PWT, or (b) the upper/lower end of the von Frey hair set was reached before a positive/negative response had been obtained. We planned to exclude from the study any rats that did not develop mechanical hypersensitivity (>50% reduction after model establishment), and two animals had to be excluded based on this criterion. To minimize the variability in behavioral outcomes, animals were trained for 3–5 days before baseline data were obtained (Chen et al., 2019). Additional PWTs were also measured within 30 min to 2 h after fat emulsion or FA administration on the day of operation and on postoperative day 1 (POD1) and POD2; all tests were completed between 8:00 and 16:00 h. The researcher who conducted the PWT tests was blinded to the group allocation.

### Animal allocation and drug administration

After successfully establishing the incision pain model, 40 SH rats were randomly divided into a vehicle (H0) group that received medium/long-chain fat emulsion (Huarui Pharmaceutical, Wuxi, China) at 0.5 mL/d, and three groups that received FA (Beijing Tide Pharmaceutical, Beijing, China) at different doses: the low-dose (H12.5) group received 12.5 mg/kg/d; the medium-dose (H25) group, 25 mg/kg/d; and the high-dose (H50) group, 50 mg/kg/d (n = 10 animals in all groups). Another 40 WKY rats were included as controls and allocated into the four analogous groups of vehicles (C0, C12.5, C25, and C50). All rats were given fat emulsion or different FA doses via tail vein injection for three consecutive days beginning from the day of operation. The daily dose was administered twice at a 6-h interval. All human equivalent doses were calculated using body surface area conversion factors (6.2 × the human dose) (Reagan-Shaw et al., 2008).

### Renal function test

On the day before surgery, POD2 and POD7, 1 mL of tail vein blood was collected and then centrifuged (2,000 *g* in 15 min), and the serum was separated and stored at −80 °C prior to analysis. Urine (0.3 mL) was collected from each rat at the same time points. Specific kits were used to determine SCr (Sichuan Maccura Biotechnology, Chengdu, China), serum Cys C (Beijing Baiding Bioengineering, Beijing, China), and urinary NAG activity (Beijing Wantai DRD, Beijing, China) according to the protocols recommended by the manufacturer, using an automatic biochemical analyzer (Rayto Life and Analytical Sciences, Shenzhen, China).

### Renal pathology

Rats were euthanized on POD7. The kidneys were removed and immediately fixed in 10% phosphate-buffered formalin for 24 h. Transverse kidney samples were paraffin-embedded, cut, deparaffinized, stained with hematoxylin and eosin (H&E), and examined under a light microscope.

### Statistical analysis

All data were expressed as mean ± standard deviation (SD), and inter-group differences were assessed for significance using one-way analysis of variance, followed by Fisher’s least significant difference (LSD) method. Inter-group differences in blood pressure and pain behavioral test results were assessed using repeated-measures analysis of variance. Inter-group differences for the same time point were assessed using analysis of variance, followed by the LSD method for pairwise comparison. All analyses were performed using SPSS 19.0 (SPSS, Chicago, IL, United States). Differences associated with P < 0.05 were considered statistically significant.

## Results

### Baseline animal conditions before surgery

There were no statistical differences in body weight or other health parameters between SH and WKY rats before surgery, except baseline blood pressure (Table 1). Each SH subgroup exhibited higher SBP than their counterpart WKY rats (all P < 0.01), but no other significant differences were observed.

**TABLE 1:** Baseline systolic blood pressure (mmHg) in rats treated or not with flurbiprofen axetil.

Animals	Vehicle	Flurbiprofen axetil (mg/kg/d)		
		12.5	25	50
Control rats (Wistar Kyoto)	140.1 ± 2.3	141.9 ± 1.8	141.8 ± 2.7	141.0 ± 2.1
Spontaneously hypertensive rats	164.9 ± 2.1**	165.1 ± 2.4**	165.1 ± 2.8**	164.5 ± 2.0**

Data are expressed as mean ± SD, of 10 animals per subgroup. The upper row corresponds to the control vehicle, C12.5, C25 and C50 groups in the main text. The lower row corresponds to the vehicle, H12.5, H25 and H50 groups in the main text.

**P < 0.01 compared to the corresponding control subgroup.

### Medium-dose FA alleviated plantar incision-induced tactile allodynia with a ceiling effect

**FIGURE 1:**
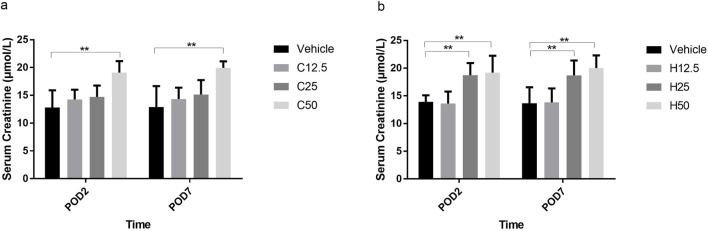
Paw withdrawal threshold (PWT) on different postoperative days (POD) after flurbiprofen axetil (FA) administration in control Wistar Kyoto **(a)** and spontaneously hypertensive **(b)** rats. Group names indicate the drug dose in mg/kg/d. Data are mean ± SD (n=10 per group). *P < 0.05, **P < 0.01.

In WKY rats, there was no difference in PWT between the C12.5 group and the vehicle group on the day of operation. The C25 and C50 groups showed similarly high PWTs that were significantly higher than those in the vehicle group on POD1 and POD2 (Figure 1; all P < 0.001).

In SH rats, the PWTs of H12.5, H25, and H50 groups were significantly higher than those of the vehicle group from the day of surgery through POD2 (all P < 0.001). The H25 and H50 groups showed similarly high PWTs that were significantly higher than those in the H12.5 group on POD1 (P = 0.022) and POD2 (P < 0.001) (Figure 1).

### FA induced an increase of SCr in SH rats

**FIGURE 2:**
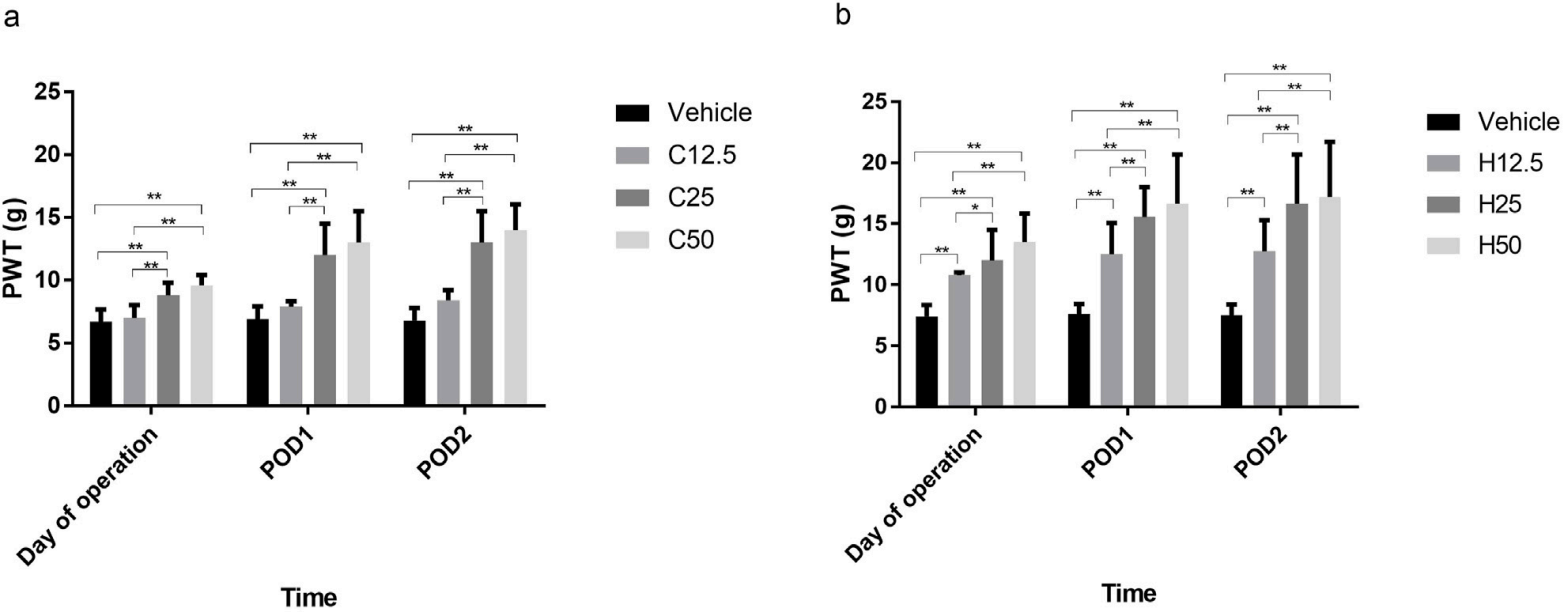
Serum creatinine concentrations after flurbiprofen axetil (FA) administration on different postoperative days (POD) in control Wistar Kyoto **(a)** and spontaneously hypertensive **(b)** rats. Group names indicate the drug dose in mg/kg/d. Data are mean ± SD (n=10 per group). *P < 0.05, **P < 0.01.

In WKY rats, SCr levels in C12.5 and C25 groups were similar to those in the vehicle group on POD2 and POD7, but SCr levels were significantly higher in the C50 group than in the vehicle group (all P < 0.001) (Figure 2). In SH rats, there was no significant difference in SCr levels in the H12.5 group compared with the vehicle group on POD2 or POD7. However, SCr levels were significantly higher in the H25 and H50 groups (P < 0.001), with the elevation more obvious in the H50 group (Figure 2).

### Cys C levels were more sensitive than SCr levels to FA administration

**FIGURE 3:**
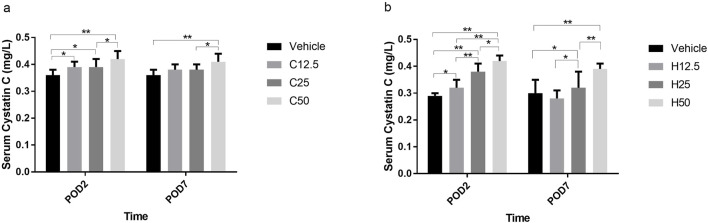
Serum Cystatin C concentrations after flurbiprofen axetil (FA) administration on different postoperative days (POD) in control Wistar Kyoto **(a)** and spontaneously hypertensive **(b)** rats. Group names indicate the drug dose in mg/kg/d. Data are mean ± SD (n=10 per group). *P < 0.05, **P < 0.01.

In WKY rats, Cys C levels were significantly higher in the C12.5, C25, and C50 groups than that in the vehicle group on POD2, but the Cys C level only increased in C50 group on POD7 (all P < 0.05) (Figure 3a). Similar changes were observed in SH rats on POD2. However, a dose-dependent increasing trend was detected in H25, and H50 groups on POD7 compared with the vehicle group (Figure 3b). This suggests that significant increases in Cys C were detectable in SH and WKY rats at lower FA doses than increases in SCr (see preceding section).

### Low-dose FA induced an increase in urinary NAG activity in SH rats

**FIGURE 4:**
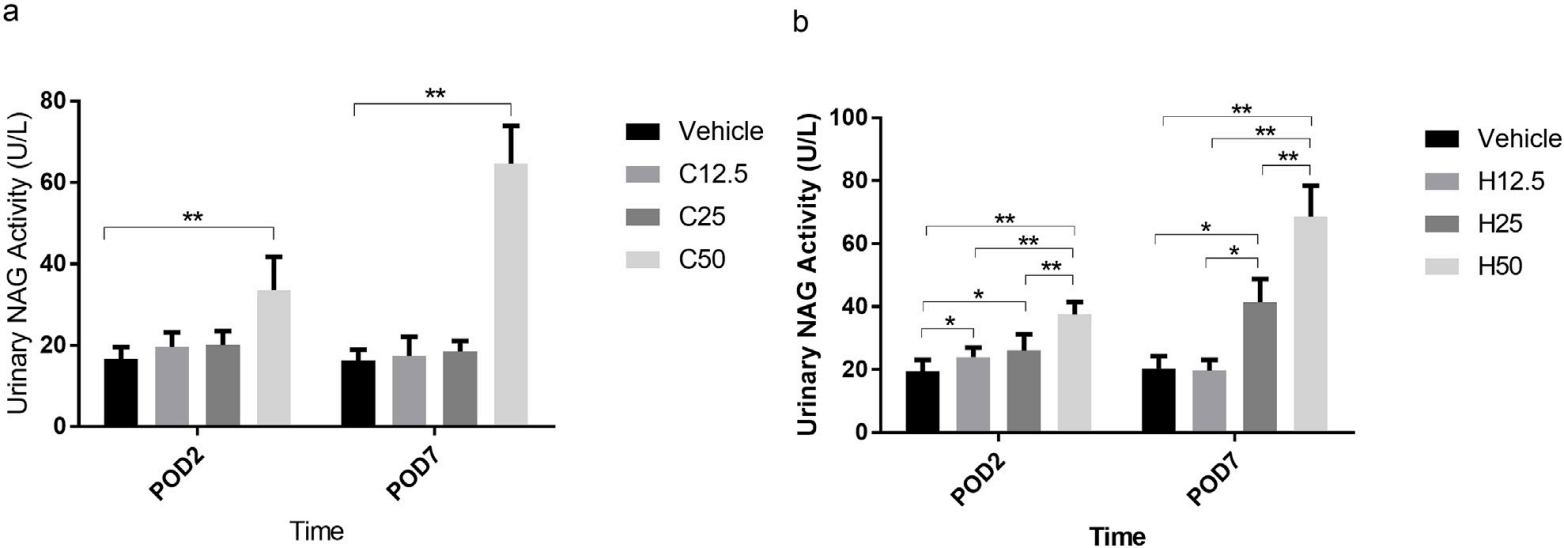
Activity of N-acetyl-β-D-glucosaminidase (NAG) in urine after flurbiprofen axetil (FA) administration on different postoperative days (POD) in control Wistar Kyoto **(a)** and spontaneously hypertensive **(b)** rats. Group names indicate the drug dose in mg/kg/d. Data are mean ± SD (n=10 per group). *P < 0.05, **P < 0.01.

In WKY rats, there was no statistical difference in NAG activity between the vehicle group and either the C12.5 or C25 group on POD2 or POD7. In contrast, NAG activity in the C50 group was significantly higher than in the vehicle group on both days (P < 0.001; Figure 4). In SH rats, NAG activity in the H12.5 group was significantly higher than in the vehicle group on POD2 (P = 0.031), after which it decreased to vehicle level by POD7. Urinary NAG activity exhibited a dose-dependent increase in H25 and H50 groups on POD2 and POD7 (Figure 4). These results suggest that low-dose FA can induce a significant increase in urinary NAG activity in SH but not WKY rats.

### Low-dose FA compromised glomerular and tubular structure in SH rats

**FIGURE 5:**
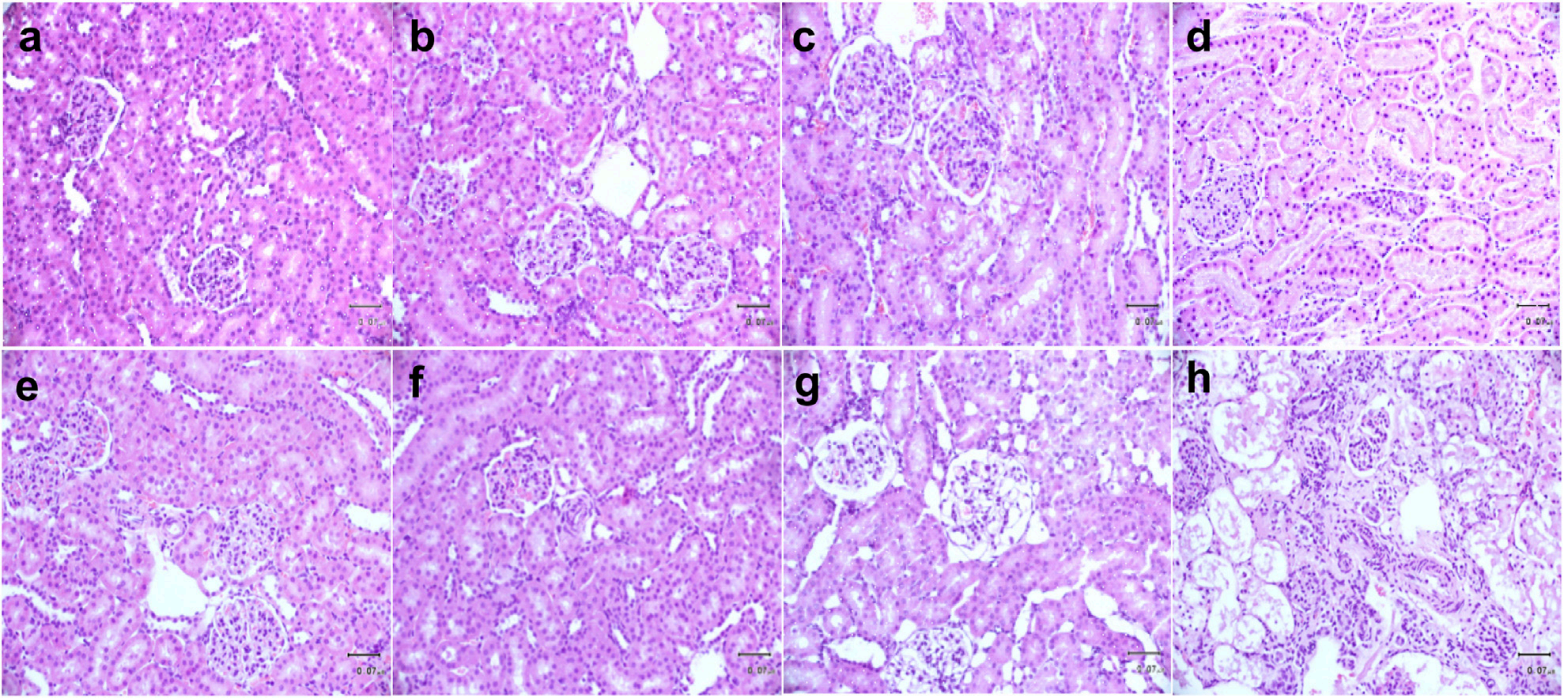
Histopathology of kidney tissue on postoperative day 7 in **(a–d)** control Wistar Kyoto rats and (**e–h)** spontaneously hypertensive rats after flurbiprofen axetil (FA) administration. Tissue sections were stained with hematoxylin and eosin. In each row, the images are shown from *left* to *right* as vehicle, 12.5 mg/kg/d FA, 25 mg/kg/d FA, and 50 mg/kg/d. Arrows indicate glomerular or tubular damage. Scale bar, 70 μm.

Kidneys showed normal glomerular structure and tubular epithelial cells in the vehicle (Figure 5a), C12.5 (Figure 5b), C25 (Figure 5c) and C50 (Figure 5d) groups of WKY rats and vehicle (Figure 5e), H12.5 (Figure 5f), H25 (Figure 5g) and H50 (Figure 5h) groups of SH rats. The H25 group, in contrast, showed glomerular atrophy, focal segmental sclerosis, stenosis or disappearance of the renal capsule, and partial vacuolar degeneration of renal tubular epithelial cells (Figure 5d). The C50 and H50 groups showed extensive autolysis and necrosis in the renal cortex, smaller residual glomerular volume, atrophy, diffuse spheroidal sclerosis, renal capsule expansion and autolysis, as well as autolysis and disappearance of renal tubular epithelial cells (Figures 5g,h). These FA-induced pathological changes were consistent with our measurements of renal function indices in the preceding sections.

## Discussion

NSAIDs are the most widely prescribed analgesics in the clinic, especially during the preoperative period. With the rapid development of enhanced recovery after surgery (ERAS), postoperative use of NSAIDs has surged. However, NSAID-associated adverse effects, including renal impairment, still pose some major challenges. In particular, their postoperative safety and efficacy are unclear in elderly surgical patients with preexisting hypertension and/or renal damage (Amatruda et al., 2021).

Likewise, although the opioid-sparing effect of FA in clinical settings is known (Yang and Hu, 2025; Wang et al., 2022), the optimal analgesic dose of FA as well as its safety are poorly understood. Here we report that medium-dose FA can effectively alleviate postoperative pain and achieve a ceiling effect in SH rats. FA can reduce glomerular and tubular function to a certain extent in both normo- and hypertensive rats. Impairment of renal function occurs much earlier after surgery in hypertensive rats than in normotensive rats, even with low-dose FA.

We observed an analgesic effect of medium-dose FA until a plateau in both SH and WKY rats. Consistent with our results, previous studies showed that several types of NSAIDs (e.g., ketorolac and ibuprofen) exhibit an analgesic ceiling, after which they offer no incremental analgesia but only increase risk of adverse events (Motov et al., 2017; Weiser et al., 2018). Similarly, a study of patients undergoing laparoscopic cholecystectomy revealed that FA used above the ceiling dose (150 mg) exerted no enhanced analgesic effect but increased the incidence of adverse reactions (Zhao and Ji, 2018). We found PWT differences between the two types of rats at different postoperative time points, which may be explained by the increased pain threshold associated with hypertension (Ghione, 1996; Graham, 2001). This possibility should be investigated in future studies.

Renal impairment is a major concern with NSAIDs, particularly at doses above the analgesic ceiling. Some evidence has suggested that NSAID-associated acute renal injury is dose-dependent (Wang et al., 2020). We found that FA caused similar dose-dependent glomerular and tubular impairments in normo- and hypertensive rats. However, low-dose FA was sufficient to cause significant increases in blood indices of glomerular and tubular damage as well as histopathological changes in hypertensive rats, but not in normotensive animals. This likely reflects the more fragile renal function in hypertensive rats. A previous large single-center retrospective study revealed FA’s effect on postoperative AKI was dose-dependent, and using a low dose of FA (50–100 mg) perioperatively was even significantly associated with a decreased incidence of postoperative AKI (Wang et al., 2020). However, when the dose reached ≧250 mg, FA significantly increased the incidence of AKI, but the middle dose of FA (100–250 mg) had no impact on AKI. These conflicting results may be attributed to the subjects *per se*, comorbidity and other baseline and intervention metrics, which merits further investigation.

The major mechanism of action of NSAIDs is inhibition of cyclooxygenase (COX), which blocks the biosynthesis of prostaglandins (PGs) that promote pain and inflammation. There are two COX isoforms, COX-1 and COX-2, with similar biological activities but distinct tissue expression and regulation. COX-1 is constitutively expressed in mesangium, epithelium and endothelium, specifically cortical and medullary collecting duct cells, and it regulates the synthesis of the vasoconstrictor thromboxane A_2_ (TXA_2_). COX-2 is produced by vascular and tubular structures in the kidney, and mediates the production of the prostaglandin vasodilators PGE_2_ and PGI_2_. These two COX isoforms maintain the homeostasis of glomerular and tubular function (Hetu and Riendeau, 2005; Theilig et al., 2006). The FA used in the present study is a non-selective NSAID that exerts similar inhibitory effects against COX-1 and COX-2, and thus has an effect on both glomerular and tubular functions. PGI_2_ and PGE_2_ play a major role in the maintenance of renal perfusion and the regulation of electrolytes. Intravenous infusion of PGI_2_ can increase renal blood flow in healthy individuals (Nielsen et al., 1997), while PGE_2_ promotes the secretion of renin from paragonadal cells (Wagner et al., 1998). Long-term hypertension can lead to renal arteriosclerosis, resulting in chronic glomerular and tubular damage. This, coupled with excessive activation of the local renin-angiotensin system, ultimately leads to renal parenchymal ischemia, atrophy, fibrosis, and even necrosis. To protect against this injury, SH rats upregulate COX-2-mediated production prostaglandins (Khan et al., 2019; Teixeira-da-Silva et al., 2019). These considerations lead us to conclude that use of FA or other NSAIDs weakens the already compromised prostaglandin-based renal-vascular regulation, increasing the risk of renal ischemic injury and acute tubular necrosis (Schneider et al., 2006).

In our study, we were able to detect glomerular impairment in normo- and hypertensive rats earlier using Cys C than the widely used SCr index of renal function. The association of Cys C with estimated GFR and drug pharmacokinetics has been validated in clinical contexts including transplantation, corticosteroid treatment, disorders of compromised muscle mass or cachexia, malignancy, and obesity (Teaford et al., 2020). Therefore, we propose Cys C as an alternative or complement to SCr for detecting FA-associated postoperative glomerular dysfunction in high-risk patients, such as those with hypertension.

Our study presents several limitations. First, the relative extents of inhibition of the different COX isoforms may lead to diverse effects on blood hypertension (Khan et al., 2019), with potential impact on renal function. We were unable to assess this in our study because we did not measure blood pressure postoperatively. Second, we included only three doses of FA for three consecutive days, so future investigations are needed to explore other doses and time points. Third, our results on animal models cannot be directly translated to the clinic. Instead, future clinical studies should be conducted to identify the optimal dose of FA that achieves the maximal analgesic effect while avoiding renal function impairment.

## Conclusion

Despite these limitations, our animal study provides evidence that FA can be effective at improving postoperative pain with a ceiling analgesic effect at a medium dose of 25 mg/kg/d in rats. At the same time, FA can compromise renal function in a dose-dependent manner, which may be detectable at an earlier stage using Cys C rather than the more widely used SCr. This FA-induced renal damage may occur at lower FA doses in a background of hypertension. Our preclinical study provides a theoretical basis for the clinical use of FA in surgical patients undergoing postoperative analgesia, and it suggests factors to consider when optimizing the drug’s efficacy and safety.

## Data Availability

The original contributions presented in the study are included in the article/supplementary material, further inquiries can be directed to the corresponding author.
